# Psychological meanings reported by patients with Graves’ Disease in hyperthyroidism but without ophthalmopathy about their quotidian life: A qualitative study conducted in a Brazilian university specialized outpatient service

**DOI:** 10.1192/j.eurpsy.2024.1032

**Published:** 2024-08-27

**Authors:** L. S. Valladão, F. S. Silva, C. F. Casagrande, L. M. Guerra, D. E. Zantut-Wittmann, E. R. Turato

**Affiliations:** Lab of Clinical-Qualitative Research, State University of Campinas, Campinas, Brazil

## Abstract

**Introduction:**

To handle well clinical treatments, it is crucial to know the expectations of patients who seek help. We need to ask ourselves: how do patients interpret subjectively their diagnosis, treatments, and self-care? Medical Psychology brings us theories for this understanding. Grave’s Disease is an autoimmune disorder, a form of hyperthyroidism with a goitre, affecting also the eyes and the skin, as well as emotional manifestations. Weight loss, sometimes psychologically welcome, although due to a disease, can mean a psychoanalytic secondary gain. So, the medicine that leads to clinical improvement can be taken with ambivalence and bad adherence to treatment. It is important to differentiate between disease, a scientific entity explained by the clinical professional, and illness as a patient’s subjective perception of an un-health.

**Objectives:**

To understand psychodynamically the fantasies, desires, and views related to Graves’ Disease as reported by patients in hyperthyroidism but without ophthalmopathy interviewed at an endocrinology-specialized outpatient clinic. (in the EPA-2023, it was presented the qualitative results of a sample in hyperthyroidism, with ophthalmopathy, studied at the same service).

**Methods:**

Clinical-Qualitative Method designed by Turato. Data collected through Semi-Directed Interviews with Open-ended Questions in-Depth; and Field Notes, transcript fully. Treated by the Seven Steps of Clinical-Qualitative Content Analysis of Faria-Schützer, using psychodynamic concepts from Balintian Medical Psychology. Although we have extracted categories that permit us interesting discussions, we intend to close the sample (through the information saturation criterion by Fontanella) when we obtain other categories. The interviewer, a male psychologist, is the first author. The findings are validated by peer-reviewers of the Lab of Clinical Qualitative Research of the State University of Campinas.

**Results:**

Three categories were chosen for this presentation: 1) “An atomic bomb in my life”: How drastic changes of a hormonal disease re-symbolize the patient’s life; 2) “I didn’t think the thyroid did that much”: the disease seen as a metaphor in a psychological blaming language to own disease and to himself as a sick person. 3) “I have so much medicine!”: a mode of referring to treatment that would justify an undisciplined use of medications.

**Conclusions:**

Our findings can help clinical professionals to have a better understanding of some psychological meanings which have sense in the patients’ conscience, often not verbalized clearly in the conversation, and so to handle better the patients and relatives. In this way, it can reduce the patient’s resistance to recommended treatment, as well as encourage the clinical team to construct empathy with them.

**Disclosure of Interest:**

None Declared

